# Responsible Governance for a Food and Nutrition E-Infrastructure: Case Study of the Determinants and Intake Data Platform

**DOI:** 10.3389/fnut.2021.795802

**Published:** 2022-03-23

**Authors:** Lada Timotijevic, Indira Carr, Javier De La Cueva, Tome Eftimov, Charo E. Hodgkins, Barbara Koroušić Seljak, Bent E. Mikkelsen, Trond Selnes, Pieter Van't Veer, Karin Zimmermann

**Affiliations:** ^1^School of Psychology, University of Surrey, Guildford, United Kingdom; ^2^School of Law, University of Surrey, Guildford, United Kingdom; ^3^Javier de la Cueva and Asociados, Madrid, Spain; ^4^Jožef Stefan Institute, Ljubljana, Slovenia; ^5^Department of Geosciences and Natural Resource Management, University of Copenhagen, Copenhagen, Denmark; ^6^Wageningen Economic Research, Wageningen University and Research Centre, Wageningen, Netherlands

**Keywords:** food consumer behavior, food consumer choice, data quality, interoperability, standardization, big data, ethical, machine learning

## Abstract

The focus of the current paper is on a design of responsible governance of food consumer science e-infrastructure using the case study Determinants and Intake Data Platform (DI Data Platform). One of the key challenges for implementation of the DI Data Platform is how to develop responsible governance that observes the ethical and legal frameworks of big data research and innovation, whilst simultaneously capitalizing on huge opportunities offered by open science and the use of big data in food consumer science research. We address this challenge with a specific focus on four key governance considerations: data type and technology; data ownership and intellectual property; data privacy and security; and institutional arrangements for ethical governance. The paper concludes with a set of responsible research governance principles that can inform the implementation of DI Data Platform, and in particular: consider both individual and group privacy; monitor the power and control (e.g., between the scientist and the research participant) in the process of research; question the veracity of new knowledge based on big data analytics; understand the diverse interpretations of scientists' responsibility across different jurisdictions.

## Introduction

Big data provides immense opportunities to radically alter the way in which science is done, fostering cross-fertilization between disciplines, and providing connectivity between disparate data-sets. There has been a huge surge in recent years of initiatives to develop structures and networks that foster data gathering, connectivity and large data analytics to ensure advancements in core areas of science. Distributed computing infrastructures—commonly known as e-infrastructures—have been created that provide researchers shared access to large data collections enabled through advanced Information Communications, large-scale computing resources, and high-performance visualization (1). Research e-infrastructures have received considerable EU funding, with over 20 billion Euros currently being invested for their development (2). However, the scientific domain of food consumption and its relation to health and sustainability, is not yet facilitated by a well-supported research infrastructure (RI). In the area of food, there are well-established research infrastructures in the omics and nutritional systems biology domain [e.g., ELIXIR^1^], medical sciences domain [e.g., clinical research: ECRIN ERIC^2^; translational medicine: EATRIS ERIC^3^, biobanking: BBMRI, ERIC^4^], and health research is relatively well funded by the public and private sector (3). However, it is increasingly recognized that the focus on diseases brings a curative bias and, as the COVID pandemic has amply demonstrated, prevention is a crucial aspect of well-functioning social and health care (4). Similarly, agriculture has traditionally been a domain for public investment in research, with the emergence of research infrastructures in agri-ecosystems such as Analysis and Experimentation on Eco-systems^5^. Indeed, in the food domain, environmental issues are paramount. But there is increasing recognition that the current global challenges associated with food and environmental sustainability cannot only be solved within a productivist paradigm and the associated technical solutions, instead, that we need to explore the consumption process and its interaction with the production (5–7).

The case for a dedicated food nutrition e-infrastructure has been made in the past several years with the emergence of a network of scientists set to develop Food Nutrition Health Research Infrastructure^6^. A fundamental part of this international initiative is the development of an e-infrastructure that enables innovative science in the domain of dietary determinants and intake. Using ICT and new technology such as smart phones, APPs, sensors, internet of things and big data offers new ways of exploring food consumption in the context of the food chain. To harness these new technologies and address this gap, the Determinants and Intake (DI) Data Platform has been forged from the work of two European projects – EuroDISH (2012–2014) and Richfields (2015–2018). The DI Data Platform emerged in recognition that the food chain and consumer food choices are of direct relevance to public health, prevention, health promotion, environmental sustainability and socioeconomic impacts of the food system, but the domain of food consumption has been omitted from research funding (3, 8). Intensifying the research in this domain would therefore be necessary (9).

The focus of the current paper is to examine how to develop responsible governance of a food nutrition e-infrastructure using the case study Determinants and Intake (DI) Data Platform. The proposed DI Data Platform that is being developed will ostensibly utilize big data in promoting research and innovation in this domain. Hereby rests the challenge of how to develop responsible governance that observes the ethical and legal frameworks of big data research and innovation, whilst simultaneously capitalizing on huge opportunities offered by big data in food nutrition and health research. To reach this ambitious goal and to counteract some of the reservations that actors traditionally have about big data, agreements and consensus among a broad range of stakeholders is needed and as a result a fair and accepted governance structure is necessary.

This paper first describes the unique features of big data and the broad challenges for ethical governance it poses when harnessed within a research e-infrastructure. Following the description of the vision and mission of DI Data Platform, we address the core challenges for the development of responsible governance with a specific focus on four key governance considerations: data type and technology; data privacy and security; data ownership; and institutional arrangements for ethical governance. The paper concludes with a synthesis of ethical challenges linking these to the responsible research governance principles that may help the development of the research e-infrastructures, and in particular, highlight the relevance of Responsible Research and Innovation (RRI) as a form of meta-responsibility that brings together ethical and legal aspects of governance under a single framework. RRI aims to “shape, maintain, develop, coordinate and align existing and novel research and innovation-related processes, actors and responsibilities with a view to ensuring desirable and acceptable research outcomes” (10).

## Ethical Challenges of Big Data in Science: Data, Data Processing and Data Management

The meaning of big data has been widely discussed (11–20) as efforts have been made to delineate the concept. Fothergill et al. (11) summarize the literature that has attempted to define and explain big data. Big data is often described as “large volumes of high velocity, complex, and variable data that require advanced techniques and technologies to enable the capture, storage, distribution, management and analysis of the information” (p. 11) (21). It's value to research is derived from its specific properties: it is indexical in nature, relational, flexible, scalable, re-purposable, continuously updatable and easily removable from the context of data collection (3).

The sheer scale of big data poses ethical, legal and societal challenges to e-infrastructures. There is a growing literature concerned with addressing these as part of the projects for research e-infrastructure building workable data governance frameworks [e.g., (2, 11)]. Common to most of this literature is the realization that responsible research e-infrastructure governance is a matter of contextualized and deeply embedded decision-making that departs to a large extent from the principles of the conventional research ethics and governance enshrined in professional practice and law. Governance specifies how decision-making within an organization should be structured and implies allocation of responsibility (22), in terms of who is responsible for what and under what conditions. Responsibility toward an individual participant has been the main focus of legal frameworks developed to regulate traditional scientific processes. For instance, in the EU, the General Data Protection Regulation (23) is developed with an explicit remit to protect individual^7^ privacy rights, whilst at the same time to remove the obstacles to flows of personal data within the Union through harmonization of the law across the member states. Outside of Europe, the regulation is seemingly much more fragmented—for instance the US has only recently introduced Information Transparency and Personal Data Control Act^8^, which protects personal information and institutes the Federal Trade Commission responsible for the development and oversight of the requirementsts for collecting, processing, storing and sharing sensitive personal information. The bill however does not include the right of an individual to access, correct or delete the data stored about them, which is included within the EU GDPR.

Big data has revealed new challenges for our conceptualization of researchers' and scientists' responsibilities when utilizing big data. Researching with big data makes it incumbent upon researchers to re-think their own responsibilities vis-à-vis both the participants but also society at large. This has resulted in the explicit need to broaden out the scope of responsible research governance to include considerations of diverse data types (not just human and animal data), analytical processes, group protections and long term implications of research (3). It has provided new challenges to considerations of the rights of the research participants and the rights and responsibilities of researchers.

This paper will highlight these challenges through the case of developing DI Data Platform. In the sections that follow we present the core considerations that have informed the development of the responsible governance approach for this e-infrastructure, and which are analyzed in light of the principles of RRI.

## The Case of Determinants and Intake Data Platform

A conceptual design of DI Data Platform was built on the vision of a Food, Nutrition and Health Research Infrastructure (hereafter: FNH-RI), which would connect the data and science in the domains that link food consumption, sustainability and health. It was first proposed by the EU project EuroDISH [www.eurodish.eu; (9)], which defined the main pillars of the proposed FNH-RI: **D**eterminants of food choice (why do we buy and eat what we eat?), **I**ntake of food (what do we actually eat, when, where and how?), **S**tatus of the body (how overweight, obese are we?) and **H**ealth. One finding of the EuroDISH project was the need for better data in the pillars of Determinants and Intake where there is notable absence of standardized concepts, methods and tools for data collection, and no international data depositaries. This led to an EU-funded project RICHFIELDS (acronym for Research Infrastructure on Consumer Health and Food Intake for E-science with Linked Data Sharing) with an explicit remit to prepare a design for a Determinants Intake (DI) Data Platform. The idea was to implement this platform into a larger FNH RI, with a unique infrastructure providing data services (“DATA”), facilities and tools (“FACT”), and training, education and dissemination (“TED”) to scientists based on standardized collection and integration of data by a DI “Richfields” Consumer APP drawn from consumer-donated data; as well as research and business data repositories, drawn from public research institutions, business and non-governmental organizations. The provision of these scientific services will ultimately enable policymakers, NGOs, food industries, SMEs, farmers and consumers to make more responsible decisions.

**Figure 1:**
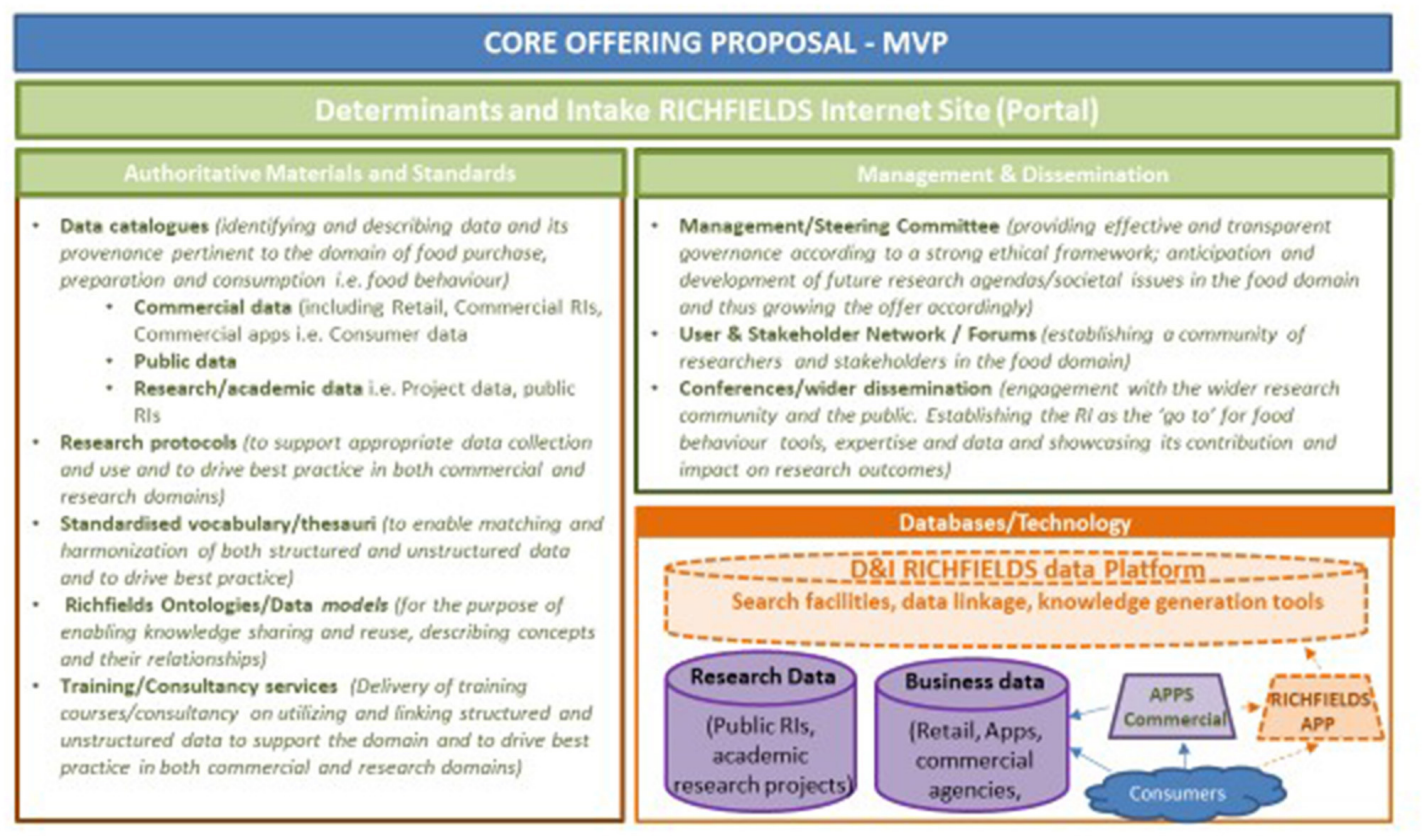
Core offering of DI “Richfields” Data Platform - a minimum viable product.

The DI Data Platform combines different types of food consumer science data: consumer-generated data, mostly real-time and *in situ* (e.g., food consumption data generated via APPs); business-generated data (e.g., sales data, food composition data); and research-generated data from research laboratories, experimental facilities and from existing and developing RIs (24). The DI Data Platform is summarized in Figure 1.

Ultimately, the DI Data Platform will enable research with real impact on our current food system, addressing, through the use of innovative technologies and big data, the current challenges of consumption and production within the food system. Table 1 summarizes the areas of research and the associated food system domains the DI Data Platform will address.

**Table 1:** Examples of research and innovation domains of food nutrition and health research that can be supported by determinants and intake data platform.

		**Scientific research domains (9)**				
**Food system**
	Building blocks of the food system	Food chain: food security, safety, quality, environmental sustainability	Consumer behavior: Determinants and intake of foods and nutrients	Consumer health: Status and function of the body, up to risk for health and disease	Development into coherent research domain	
	Consumers, foods and diets	Food reformulation toward energy-poor and nutrient- rich food supply	Innovative assessments by apps, sensors, wearables; ambulatory monitoring Communication of environmental sustainability of food supply to consumers and stakeholders.	Sensors and wearables for e.g., heart rate monitoring, blood glucose, lipids, etc.	Food Consumer science	
		Sustainable products and replacers of animal proteins design according to consumer needs	Food choices, preferences, hunger and satiety, behavior.	Biomolecular, (bio) chemical mechanisms and (patho-) physiological disease pathways for major chronic diseases and nutritional deficiencies.		
		High (nutritional) quality foods for acceptable prices	Interoperable EU-nutrition surveillance system, incl physical activity and psycho-social determinants.	Personalized nutrition for clinical settings and high risk groups; standard for dieticians, available to citizens.		
		Data from social surveys, nutritional epidemiology and community interventions are interoperable and link to pan-EU multicentre studies; they include health and safety issues and can line up with other food systems outcomes (social, environmental, economic).				
	Consumers and the food environment	Portion sizes and labeling	Communication of health and nutritional quality of food products in food environments to consumers	Using big data to link consumer behavior and health risks.		Food systems science
		Access and affordability to foods for all socio-economic groups	Communication of environmental sustainability of food products and food chain to consumers and stakeholders in food environment	Precision nutrition – linking genetics, food environment and behavior.		
	Consumers and food supply chain	Standardized and valid LCAs on GHGe, LU and FFU. From farmgate, regional distributed center, consumer, waste.	Recipes/food composition enriches FCDBs	Food producers, retailers, restaurants and catering can evaluate the health and sustainability of their products, recipes and menus through transparent and standardized procedures, benchmarking their corporate responsibility	Agri-food science	
		Closed nutrient cycles, e.g., for carbon and nitrogen, eutrophication and acidification minimized.				
		Sourcing of commodities respects social justice, equity, animal welfare and biodiversity	Alternatives for animal protein	Production quantities, nutrients and food processing meet health requirements.		

## Case Study Results: Developing Responsible Governance for DI Data Platform

Responsible research governance of the DI Data Platform has been a fundamental part of the data platform design. For information about our approach to the platform development, please refer to the published articles (25–28). The sections below address responsible research governance in the context of data type and technology; data ownership; privacy and security; and institutional arrangements.

### Data Type and Technology Relevant to DI Data Platform

Development of responsible governance requires clear understanding of the food consumer science data being used, its purpose and value, technologies for data harvesting, and the social and legal implications of their use.

The scientific relevance of the proposed data platform is dependent on the diversity of data available to it and these include:

- **Research data** from other research infrastructures, laboratories and experimental facilities. This is mostly structured data.^9^
- **Business data** (e.g., data from retailers, public procurement companies, statistical institutions and market organizations). This can be structured, semi-structured^10^ and unstructured data.^11^
- **Consumer-generated data** from APPS (smartphone and tablet applications) and sensors. This is mostly unstructured data.

The data types relevant to the DI Data Platform can be referred to as reference data (e.g., food composition data), observational data (e.g., food intake data, physical activity), or data that is transformed into output data (e.g., nutrient intake, dietary patterns). Each of these types can relate to three key domains of human behavior that are relevant to food intake: purchase, preparation and consumption, each presenting particular issues and considerations for the DI Data Platform governance.

#### Research Data

Linking between research data held within the **existing RIs (e.g., ELIXIR**,^12^ **ECRIN ERIC)** in the food and health domain and the DI Data Platform is possibly the most accessible form of research data. However, the development of a DI Data Platform ontology and the harmonization of entities, food classification and description systems is fundamental to facilitate future data access/exchange between existing and new RIs. The development of authoritative materials and standards is also fundamental in order to establish best practice and to help shape the research community moving forwards thus making future data sharing activities easier. This will to a large extent depend on developing a community of experts in the field engaged to provide best practice guidelines and standards for data ontologies and structured data capture. **Laboratories/Data facilities (public and private)** also represent an opportunity to harvest data for research—of the 39 labs and facilities in Europe involved in consumer research in the food and health domain, many collect the data that is proprietary and typically not formatted, standardized or stored in a manner conducive to sharing outside the original purposes of the research study undertaken. In addition, the diversity of data-generating devices including video and audio results in a wide variety of data types increasing the difficulty of *post-hoc* data integration. The task of transforming data into information that can be integrated can be solved using various technologies, depending on the type of data. For instance, information extraction based on Natural Language Processing (NLP) can be used for dealing with textual data that is one the major sources of data (e.g., from literature, media and social media). Another modern technology is Deep Learning (DL) for automatically recognizing entities (information) from images. Transforming information into knowledge requires Machine Learning/Artificial Intelligence (ML/AI) techniques, such as Named Entity Recognition (NER) and Representative Learning for describing information in a form of knowledge (e.g., as a knowledge graph). Each of these technologies, however, carry a potential risk of introducing errors as a result of improper digitization or storage of information, and the errors introduced due to the geographic and cultural differences in the meaning of the data and its interpretation (29). Furthermore, by its very nature, ML can perpetuate the existing biases in the data, mis-representing aspects of reality.

Similarly with structured public research data, it would be paramount to harmonize Standard Operating Procedures (SOPs), data management protocols, including calibration/standardization protocols and improved approaches to obtaining ethical consent at the outset of the studies for future sharing with the wider research community. Processes and procedures need to be in place that would provide a full account of the nature of the ML/AI and AI used in the case of DI Data Platform data linking and extrapolation, reflecting on the consequences of the actions enacted by algorithms, on a case-by-case basis. This will be an explicit focus of the DI Data Platform organizational and data governance.

#### Business Data

Here, the ICT landscape is fast-paced, driven by an increasing number and connectivity of mobile devices used by consumers, and cheaper and better sensors. Table 2 gives an overview of modern IC technology either being used or with the potential for future data collection. It is clear that the DI Data Platform will be flexible enough to be able to respond to this dynamic ICT environment, however, careful consideration is needed on a case by case basis about the extent to which the data captured is reflective of the proposed research concepts and the assumptions underpinning the ML algorithms utilized in business and the DI Data Platform, to ensure that the data captured and linked is of sufficient quality to be treated as a useful variable for the DI Data Platform. Data collection may be significantly impacted by business purpose (e.g., policies to control suppliers or for organic procurement) which may limit the potential usefulness of the data for scientific purposes within the proposed DI Data Platform. Re-purposing of data needs to be carefully scrutinized and controlled such that ethical compliance with the original participants' consent is always maintained. Appropriate meta-data must be assigned to data such that the possibility of non-compliant sharing from either a legal/ethical or data owner requirement is eliminated.

**Table 2:** Review of ICT used by retail and market research organizations.

**Sector**	**Type of technology**	**Data capturing technology**	**Devices facilitating data capture**	**Type of data collected**	**Case studies**
Retail	Consumer location sensing technologies	Geo-fencing	Smartphones, GPS-devices	Location data involving a location-sensitive device (eg. smartphones with GPS)	RetailNext (Aurora, Mobile Engage), Euclid (Traffic, Insight), Shopkick (shopBeacon), Brickstream (Brickstream 3D+), Axper (3D vision, Sentinel), PathTracker
		Wi-Fi	Smartphones, tablets	Location data of smartphones connected to Wi-Fi	
		Bluetooth low energy (BLE)	iBeacon-compatible transmitters, smartphones	Proximity data to Bluetooth beacons of enabled smartphones	
		Visual systems	Analog or IP cameras, infrared cameras	Visual tracking data	
		RFID technology	Smartphone RFID reader, RFID sensors	Consumer real-time product choice and purchasing data. Aggregated shopper tracking data to determine shopping speed, purchasing speed, and geography of trips.	
		Combination of technologies mentioned above	Several sensors available that combines different data capturing technologies. E.g., Aurora from Retailnext combines video technology with BLE and WIFI.		
	E-commerce and m-Commerce	Online analytic tools for personal computers	Smartphone, personal computer, tablet	Web browsing patterns and online shopping patterns (Cookie data), online purchasing data	Adobe marketing cloud (Adobe), Virtual stores (Walmart)
		Online analytic tools for mobile devices	Smartphone, personal computer, tablet	Mobile phone data	
	Social media			Social media sentiment analysis data	Kellogg's tweet shop
	Point of sale technologies	Barcode technology	Digital barcode scanner, Smartphone barcode app (mobile point of sale), self-service checkouts, tablets, NFC tags	Consumer grocery shopping data	GfK ConsumerScan "Mini-Danmark, Mobile Point-of-Sale (SCANDIT), NFC tags in Casino supermarkets (France)
		Other point of sale hardware	Payment terminals, weighing sensors, cash registers	Amount owed, weight, money transactions	
		Cloud based Point-of-sale software	Uses data from devices mentioned in barcode technology and other point of sale hardware		Epos Now, Lightspeed Retail, Revel Systems, Lavu iPad POS
		Traditional point of sale software	Uses data from devices mentioned in barcode technology and other point of sale hardware (except smartphone barcode scanners)		AIMsi, AmberPOS, RetailSTAR
Market research organization	Automated voice response and voice recognition	Interactive voice response survey	Touchscreen, freephone, post-call transfer to survey line, computer aided telephone interviews, web, email and SMS	Consumer feedback on product purchased and used	Vision OneTotalRecall
	Digital observation and video	Digital diary and video recording	Webcam, smartphone, tablets, video camera, or some other type of digital audio/video recording device.	Consumer can either speak into the camera to describe a situation or feeling, or can take us on a tour, so to speak.	Olinger digital video diary
	Geo-location	GPS technology	Smart phone using apps with image, video capturing and survey questionnaire and integrated location	Photograph and record in-the-moment data in a specific location.	SSI's mobile QuickThoughts® 2.0 app. Geo-Intercepts app with features such as: GeoValidation, GeoIntensity and GeoNotification®.
	Neuromarketing research	Neuromarketing techniques	Smart phone, tablet and laptops using facial recognition and other neuro analytics software	Captures the expressions and emotions people exhibited toward using a product	Face Reader- Noldus IREACT and eye tracking- one vision

#### Consumer Generated Data

Whilst we typically talk about data collected via APPS and sensors (e.g., Fitbit) as being consumer-generated, in reality, unless the data is being shared directly from the consumer to the DI Data Platform, this type of data must also be considered business data. There are three domains of behavior that could be relevant for the platform: purchase, preparation and consumption. There are a number of limitations associated with the APPS collecting this data from the consumer, as summarized in Table 3.

**Table 3:** Potential opportunities and associated limitations for the scientific use of purchase, preparation, and consumption consumer-generated data.

**Domain**	**Potential opportunities**	**Limitations**
Purchase	• Inferences about the trends at the population level linked to purchase intention/food spend etc • Trends linked to C2B interactions (which retailers/restaurants/outlets are most visited) • Trends in how preferences in different food groups /products are shifting i.e., atttitudinal changes re purchase intention	• Cannot directly link to an individual purchase • Cannot directly link to the individual's consumption • Cannot identify the unit of analysis (i.e. does the data refer to the individual or household?)
Preparation	• People's search behavior online • Trends in recipe generation • Trends in social networking facilitated by food preparation knowledge/recipe sharing etc.	• Links to individual preparation behavior • Cannot directly link to purchase or consumption at an individual level
Consumption	• People's individual food intake profiles • Understanding of habitual food consumption behaviors across groups of interest	• Quality/completeness of the underlying food composition databases questionable • Quality and completeness of the self-reports through diet intake/physical activity APPS • Level of detail of the estimated food composition values is low, with APPS typically focusing on energy and macronutrients. • Lack of information regarding the procedures for estimating portion sizes • High prevalence of behavioral change objective which might pose a barrier toward a better understanding of the real determinants of food consumption behaviors as well as the ability to provide an unbiased insight in peoples' habitual food consumption behaviors

From a scientific perspective, the unknown quality and validity of the food composition databases used to underpin these APPS and the non-standardized procedures for portion size estimation means that conclusions with respect to the relationship between food consumption and nutrition-related diseases may be limited. Detailed research on the associations between specific nutrients and health outcomes may also be limited since majority of APPS in this domain focus only on energy and macronutrients.

Consumer-generated food purchase, preparation and consumption data are not typically collected in isolation from other potentially relevant data. A vital source for better understanding the possible drivers and barriers for people's food purchase, preparation and consumption behavior is likely to come from associations between these data and other relevant social, health and lifestyle data. This undoubtedly has the potential to give a more valid picture whereby different data sets corroborate each other to create a fuller, more accurate picture overall and the interconnectedness of APPS/tools now presents new opportunities to further enrich the food-related data from external sources. For example, it may be useful to gain domestic food purchase, preparation and consumption data from dedicated APPS and link this with health and lifestyle APPS for an individual. This combined data could be further enriched with demographic, situational and social context data collected through APPS such as Facebook, Twitter, TikTok, Instagram. However, the issue of ML-induced errors, biases and breeches of rights—e g., to privacy or human autonomy—are at the forefront of this endeavor. The extent to which users would find this interlinkage acceptable and be willing to share this type of extensive data with the proposed data platform will need to be carefully considered and governed, as will be discussed below. Due to the lack of available legal documents related to the terms and conditions and privacy statements linked to various APPS, there is often insufficient information available to assess the terms users must accept in order to use a service and the ways in which each APP gathers, uses, discloses, and manages their users' data. Hence, the legal limitations, organizational restrictions, confidentiality and privacy concerns related to collection, integration and dissemination of this consumer-generated data remain difficult to navigate other than on a specific case by case basis/detailed exploration with each individual APP of interest.

In short, the variety of data sources potentially involved and the varying levels of consent they carry with them present significant challenges to the open access vision of the DI Data Platform. This is especially important in a public-private business model scenario when there are often differing drivers and a different set of guiding principles in terms of ethics. This would require a fully transparent governance structure where the roles and responsibilities within it are well-defined, and which allows for an on-going, cross-sectoral and cross-disciplinary reflexivity on the role of ML/AI, the nature of the research questions and their potential impact (positive and negative) on the challenges and limits to the accuracy and validity of scientific insights.

### Data Ownership and Intellectual Property

The DI Data Platform operates in a relational context and participates in a *data cycle* composed of three scenarios where data related activities are held: the first scenario implies collecting data, that may come from a third party or be built by the activities held by the organization (surveys, sensors, personal interviews, etc.); a second scenario exists within the DI Data Platform, composed of activities such as transforming, ordering, cataloging, analyzing or deleting data; and a third scenario, where public data dissemination or its private delivery takes place.

The traditional question of the ownership of these data is losing intensity due to the different nature of the digital domain. Hess and Ostrom (30), studied the different needs of scholars when using information and concluded that property, as understood in the material world, has a different significance when applied to the digital world, and concluded that the most relevant activities are in building on a previous knowledge of where to access, extract, manage, exclude and alienate information. There is no need to “own” it in a traditional sense but rather to be able to exercise certain activities on it. Data may be owned by anyone as long as these activities are specifically allowed.

This also seems to be the view of the European Open Science Cloud (EOSC) (31), which focuses on FAIR data, where none of its 15 guiding principles includes the “ownership” of the data only on its reuse. Nevertheless, as pointed out by Labastida and Margoni (32), “Data can be covered by different layers of copyright protection making the relationship between data and copyright particularly complex”, a peculiarity that forces the need of all research organizations to avoid risks related to copyright infringement through a proactive attitude toward legal interoperability (33). In addition, the new paradigm of Open Science must be taken into account. The UNESCO's Draft Recommendation on Open Science^13^ proposed to its November 2021 General Assembly establishes as one of its key objectives adherence to Open Science thus “maximizing access to scientific knowledge and the reuse and combination of data and software, including source code, and thereby maximizing the common good achieved through public investment in scientific resources and infrastructures”.

As reviewed in Section Data type and Technology Relevant to DI Data Platform the data types relevant to the DI Data Platform come from different sources: research data, business data and consumer generated data. Practice shows that each source is associated with different terms and conditions, so it will be necessary to analyse the legal conditions that will be applicable to each dataset, hopefully in a license and not in an agreement. The tendency in ICT is to automatise to the maximum this analysis, designing tools to check interoperability (34) or standardization (35) of the licenses but in the current stage a manual check is needed.

The DI Data Platform will be able to use third parties' intellectual property works and create derivative works over them, if their license allows it, or to use immutable works due to a non-permissive license. At the same time, it will be able to create intellectual property works so, as an author, will be able to decide how to license it if the funding agent does not force specific terms and conditions.

### Privacy Concerns and Security

Adhering to the principles of Open Science poses an important question for researchers using big data of how they can harness the richness of the data whilst meeting the legal (e.g., EU GDPR) standards. The recent draft proposal for Data Governance Act 2020^14^ wishes to address the potential contradiction emerging from the clear need for data sharing for public benefit such as scientific advancement (made particularly relevant in the context of COVID-19), and the need to protect the interests of data-subjects (Article 16&17). The proposal aims to facilitate data sharing of digital data across the EU member states through the creation of new infrastructures for data sharing. It explicitly recognizes the value of data sharing for public benefit and anticipates governance measures to make it easier to re-use sensitive public sector data, including clearly specified role for data intermediaries, the role of the European Data Innovation Board with the focus on “altruistic” use of data (Article 27&28). However, whilst the need to enable easier data-sharing for scientific research in public interest is important, in practice, it is not clear what will be the process of transparently deciding on what sort of data analysis can be classified as “for public benefit”, or how to ensure appropriate oversight of data provenance. There are many semi-public organizations, private-public partnerships and affiliations, and the division between the two is not always clear in practice. It follows that without significant scrutiny on a case-by-case basis of each of the existing publicly held datasets, the data they hold is not readily useable by researchers.

The requirements imposed by the GDPR may be seen as (legally) onerous by researchers who wish to use business data and/or transfer data for research purposes. This may ultimately limit the value of any commercial data that the DI Data Platform incorporates into the proposed data platform for scientific purposes unless the issues associated with consent are fully addreessed. The proposed solution to achieve the required level of ethical consent for the re-use of consumers' data across all their APPS, is for the DI Data Platform to develop a proprietary APP that could not only act as an aggregator to link with other APPS used by an individual, but also as a means of collecting additional standardized data from a cohort of individuals that are of interest for research purposes. In this way direct consent will be obtained from the consumer for the use of their data either for general research or even for specific purposes and that consent held as meta-data within the DI Data Platform which from a governance perspective is the most desirable scenario. Providing different levels of consent options to research participants would allow them to specify exactly those stakeholder categories they are willing for their data to be shared with, in a time-limited manner. In this way direct consent could be obtained from the consumer for the use of their data either for research, policy development or commercial activities and that consent held as meta-data within the data platform, which, from a governance perspective, is the most desirable scenario.

This will be facilitated and ensured through the transparent processes of organizational governance that will ultimately serve to ensure trust of the consumers whose data is fundamental to the scientific work enabled by the DI Data Platform.

### Organizational and Institutional Governance

Organization and institutional governance of the DI Data Platform has been designed to address the aforementioned challenges with a primary aim to balance the interests of using open data vs. protecting sensitive data. The DI Data Platform is designed as a distributed research infrastructure with an independent legal status as a Foundation under the Dutch law. It will be, in fact, an intermediary for data sharing. It is managed by the Board made up of public and private stakeholder who have responsibility for ethics policy, and to safeguard compliance of all relevant laws and regulations when handling, storing, or processing personally identifiable data resulting from research and from APPS. This is built on the notion that being ethical means recognizing that privacy is “contextual” and “situational”, and that decision-making should be on a case by case basis, not reducible to a simple public/private distinction. Three advisory boards are important to the design of an ethical responsible governance: (1) the Scientific Advisory Committee (SAC); (2) Ethical Legal and Societal Issues Board ELSI); and (3) the Industry Forum (IF).

*The SAC* consists of the scientists appointed in their own right, and the Ethical Legal and Societal Issues Board (ELSI) also includes the scientists and representatives of society. They do not represent their own organization or country, and they represent a variety of disciplines relevant to the study of food consumption and ethics within the broader agenda of food systems research. Both SAC and ELSI can fill in their own vacancies and decide about the rules of engagement. An important ethical arrangement is that the DI Data Platform will be periodically evaluated by an independent visiting commission. All scientific research, especially in relation to persons and to health, has to be cleared by SAC and ELSI on the basis of a research plan before it can start. SAC and ELSI report to the highest level in the governance and have the duty to publish decisions it takes. ELSI will also advice on the protocols relating to data security, transfer of data to third countries, assessing the genuineness of a request by data users and the rules of operation in the event of requests that may be ethically dubious or questionable, data subjects' requests, and complaints procedures, and periodically reviewing privacy measures. In addition, ELSI will be making judgments whether any data sharing is permissible based on the assessments of the proposed research as being for public benefit. Crucially, the SAC and ELSI will ensure that a data protection officer is employed on a permanent basis, and, in line with the GDPR and has an oversight of all the necessary procedures, acts as a first point of call for all the interested parties, especially the data-subjects with an interest to exercise their data rights.

*Industry Forum* (IF) comprises of the industry, technology developers and other relevant business organizations. The IF will contribute to the decision-making process together with the SAC that will also include algorithmic auditing within the software-development as a means of minimizing biases and errors intrinsic to ML/AI-enabled big data research. The decisions of this forum will be subject to open and transparent rules of decision-making. The Industry Forum will need to strike a balance between many potentially competing and contradictory values such as achieving transparency, whilst protecting privacy or ensuring openness whilst respecting proprietary rights.

The most important aim of the organizational governance is to ensure a fair, transparent and inclusive data governance: the type of data and their provenance; the nature, use and limitations of ML that underpins the data brokerage within the DI Data Platform; the way in which parameters are identified and treated, and the way in which inference about the data will be performed.

## Discussion

In this paper we presented a case study exploring the decision-making in the development of responsible governance of an e-infrastructure—DI Data Platform. As illustrated above, big data is fundamentally altering the nature of research and changing the nature of the relationship between the research participant and the researcher (36). In the traditional research governance process, there is a direct relationship between the two. Research with traditional data sets is characterized by a great deal of certainty of research governance, managed through formal ethical reviews carried by university research ethics committees, on a specific type of data that is deemed to be “personal”, and which focuses on the procedures to guarantee protections of human subjects participating in research through tools such informed consent, offer of access and withdrawal of data (unless the right is waved by the data subject) and specified analytical purpose. Under the current legal frameworks [e.g., the EU GDPR (23)] it is necessary to contend with the original purpose and context of data collection and the individual implications of any alteration and re-purposing of the data.

However, as demonstrated in this case study, big data is often not collected for research purposes—for instance, retail data or APPS-generated data (which offer possibilities of new insights in food consumption research) exist to support key business operations. Furthermore, the data thus generated is not stored in discrete locations and does not allow for easy retrieval and removal of the data upon participants' request. Finally, efforts to link and integrate the data will require that scientists contend with the difficult issue of the nature and role of algorithms that enable linkages and inter-operability between diverse data sets. This poses new challenges of the meaning of participants' informed consent for data protection and data sharing under this new regime, the nature of inferences from the new approaches to data analysis and how to assess the veracity of the findings, how best to protect data-subjects' rights (e.g., rights to remove their data), and how to monitor the re-purposing and re-use of the data.

For instance, the narrow focus of the GDPR upon the risks to individual data privacy through its emphasis upon the protection of personal and special category of data and the need for de-identification of an individual does not recognize that big data research affects not only rights of individuals, but also those of groups. Identifiability is not a binary issue and the disclosure risks increase with number of data points for an individual case. A person may be anonymous yet identifiable in terms of the kind of person that he or she represents based on their behaviors, inferred attitudes, and purported identities. Identifying a person as representative of a group that can be characterized, described and located, can also result in harm – both at the personal level and at the societal level. Thus, privacy concerns are not only linked to personal data. “**Group privacy**” (18) has emerged as an important consideration specific to big data. Information about an individual, even if stripped of person-identifying information, which nevertheless links them with a group, can induce harm and pose complex ethical challenges. As highlighted in the current case study, responsible governance of big data broadens its enquiry to go beyond the narrow consideration of privacy, consent and anonymity. Instead, the meaning of data and the nature of harm it can pose must also be inferred from the way in which data is collected, the way in which it is connected to other data sets, processed, managed and controlled.

The nature of big data collection and processing fundamentally departs from the traditional scientific research. Traditional research methods in science are built on hypotheses-testing or observations based on *apriori* set of assumptions and reasonably articulated set of goals. Its primary aim is uncovering causal relationships. Big data analytics, on the other hand, uncovers patterns and correlations observed across large swathes of data and often requires that the data originally collected for a specific purpose be re-purposed and linked to other data sets that may alter its meaning encapsulated by the original aims of data collection (18). For instance, the data on people's consumption practices may not only serve the purpose of assessing consumption trends but be linked to the person's health data to provide estimates of cancer risks. This has implications for the way in which informed consent is sought, but ultimately, it removes the **power and control** over the uses of data from those who have donated it.

The processing that occurs through big data analysis is different from that of the traditional research. It is based on identifying patterns of large-scale data sets and through numerous data points it can identify patterns in the data that are not part of the initial research and scientific enquiry (18). Big data enables finding random commonalities based on incidental co-occurrence, which raises the question of **veracity of findings** because the larger the data set, the more connections can be identified through random process (18). Indeed, big data science may inadvertently create a version of the world that has little bearing on reality and provide interpretations and shape policies in a way that creates or perpetuates biases and injustices.

There is however another important ethical issue associated with the randomness of big data analytics, and it pertains to the consequences of the knowledge thus generated. One of the core issues, which will be of relevance to bio-medical and public health scientific domains, is the consequence of incidental findings, the duty of care and safeguarding. For instance, big data science can inadvertently identify certain groups which may be at more immediate risk of developing a disease (for instance, at risk to develop cancer). Whilst the ethical role of the scientist in traditional research has been defined in terms of the specific aims of the research study that made it possible to anticipate and mitigate the risks, this role is hugely complicated in the context of big data science. The possibility of **incidental findings and randomly generated knowledge** can have dual consequence: either removing scientists from any responsibility toward their research participants or broadening out the frame within which the responsibility for research is interpreted and allocated beyond the traditional scope of scientific work. The issue of what constitutes **responsible big data analytics** is a moot point, and one that requires anticipation, reflexivity and contextual decision-making, as well as greater engagement with legal frameworks other than those typically guiding the scientific process.

This brings us to the final point about the requirements for data management that must take into consideration **diverse jurisdictions**, national and regional level policies and economic realities that can be relied upon to enable smooth research governance. Big data and Open Science (37) presuppose free and interoperable/standardized flow of data across different data platforms, which may be located within diverse jurisdictions. The legal constraints of data transfers and processing in these different contexts are widely acknowledged and to an extent dealt with by the existing legal frameworks [e.g., GDPR, Chapter 5 (23)]. However, what is less often discussed is how this translates into the actual research practice in which diverse cultural norms and moral codes are in place. Whilst the Western cultural and philosophical tradition is based on the concept of autonomy, agency, self-determination and individualism, and encapsulated in the concept of human rights and respect for privacy (38), this is not necessarily the case across other cultures and philosophical traditions [e.g., Eastern European (39)]. The consequence of this may be that scientists' and data subjects' interpretation of consent and harm may vary across different cultures and therefore, that implementation of privacy laws, even if ostensibly harmonized across jurisdictions, may be variable. This poses a fundamental question of how these divergencies should be addressed within not only legal, but also ethical codes of research. Is it enough for a scientist in question to accept different readings of consent and diverse implications of autonomy, thus effectively hiding behind the (often inadequate) legal provisions in different jurisdictions? Or does a scientist have a **moral responsibility to reflect on the conditions of use of the data collected in diverse jurisdictions**, and impose own ethical standards of data use? This question has been addressed by Metcalf and Crawford (24) in their analysis of the US treatment of Big Data appearing in public domain with a problematic premise that publicly available data such as Twitter or Facebook feeds poses minimal risk to human subjects. The authors demonstrate the flaw in this position adopted by the regulator and enacted by the scientists in the US. In line with the principles of RRI, a more nuanced deliberation about the way in which the data in public domain is re-purposed for science is crucial in order to achieve responsible governance of e-infrastructures.

## Conclusions

Numerous ethical challenges are posed to scientists in the context of research e-infrastructures that connect big data across diverse regulatory, societal and epistemic regimes. The issues of privacy – both individual and group, the relationship between the researcher and the data subject, power and control in the process of research, veracity of findings and the changing nature of scientist responsibility for the generation of knowledge, represent key ethical issues that must be reflected in the context of responsible e-infrastructure governance. Addressing these requires a flexible, adaptable, responsive and transparent organizational and data governance process, open to scrutiny and in step with the technological, business and socio-political changes. RRI will form a basis of the governance of this e-infrastructure as a process guided by the principles of anticipation, reflexivity, engagement and responsiveness. Such an approach will ensure an optimal framework for responsible governance of the complex, uncertain and impactful technologies with far-reaching consequences that are likely to revolutionize the domain of food, nutrition and health (10).

## Data Availability Statement

The authors of this article conducted this research whilst working on the Richfields Project, which was used as the case study described herein. The data supporting this study should be requested from the RICHFIELDS consortium (https://fnhri.eu/contact/) and will be granted upon reasonable request.

## Author Contributions

LT contributed to the ethical design of DI data platform, synthesized the results in the article, and wrote the article. IC contributed to the data privacy and security section and reviewed the article. JD contributed to the data ownership and intellectual property section and reviewed the article. TE contributed to the data type section. CH, BK, and BM contributed to the data type section and reviewed the article. TS contributed to the organizational and institutional governance section and reviewed the article. PV was the scientific lead of the DI data platform design process and reviewed the article. KZ was the coordinator of the overall DI data platform design process. All authors contributed to the article and approved the submitted version.

## Funding

The RICHFIELDS project has received funding from the European Union's Horizon 2020 Research and Innovation Programme under grant agreement no. 654280 (www.richfields.eu).

## Conflict of Interest

The authors declare that the research was conducted in the absence of any commercial or financial relationships that could be construed as a potential conflict of interest.

## Publisher's Note

All claims expressed in this article are solely those of the authors and do not necessarily represent those of their affiliated organizations, or those of the publisher, the editors and the reviewers. Any product that may be evaluated in this article, or claim that may be made by its manufacturer, is not guaranteed or endorsed by the publisher.

## Footnotes

^1^https://elixir-europe.org/about-us (accessed October 13, 2021).

^2^https://ecrin.org/ (accessed October 13, 2021).

^3^https://eatris.eu/ (accessed October 13, 2021).

^4^https://bbmri.eu (accessed October 13, 2021).

^5^https://www.anaee.eu/about/missions (accessed October 13, 2021).

^6^https://fnhri.eu/ (accessed October 13, 2021).

^7^The EU is the process supplementing this regulation with a Proposal for a Regulation of the European Parliament and of the Council on European Data Governance (Data Governance Act) {SEC(2020) 405 final} - {SWD(2020) 295 final} - {SWD(2020) 296 final}. On 30 November 2021 the negotiators from the Council and the European Parliament reached provisional agreement on the Data Governance Act. This Act will enable reuse of certain categories of public-sector data that are subject to the rights of others, such as personal data and data protected by intellectual property rights. However public sector bodies that allow safe reuse of data need to ensure that privacy and confidentiality are fully preserved. See section *Privacy concerns and security* below.

^8^https://www.congress.gov/bill/116th-congress/house-bill/2013 (accessed October 13, 2021).

^9^Structured or relational data concerns all data, which can be stored in a relational database.

^10^Semi-structured data is a form of structured data that does not conform with the formal structure of data models associated with relational databases; however, it may have information associated with it, such as metadata tagging, that allows elements contained to be addressed (e.g., XML or JSON data).

^11^Unstructured data, such as text, PDF documents, media posts, photos, audio files etc., does not have a pre-defined data model, thus it is not a good fit for a relational database.

^12^ https://elixir-europe.org/news/elixir-launches-new-food-and-nutrition-community

^13^https://unesdoc.unesco.org/ark:/48223/pf0000374837 (accessed October 13, 2021).

^14^https://eur-lex.europa.eu/legal-content/EN/TXT/PDF/?uri=CELEX:52020PC0767&from=EN (accessed October 13, 2021).
